# Longitudinal evaluation of jaw muscle activity and mandibular kinematics in young patients with Class II malocclusion treated with the Teuscher activator

**DOI:** 10.4317/medoral.18610

**Published:** 2013-02-05

**Authors:** Maria J. Cuevas, Alberto Cacho, Jose A. Alarcón, Conchita Martín

**Affiliations:** 1 Section of Graduate Orthodontics, Complutense University, Madrid, Spain; 2 Section of Graduate Orthodontics, University of Granada, Spain; 3 ….

## Abstract

Objectives: A longitudinal study was performed to evaluate the jaw muscle activity and mandibular kinematics after Teuscher activator treatment and at 2 years after orthodontic treatment completion. Material and Methods: Twenty-seven children with Class II division 1 malocclusion were evaluated before treatment (T0; mean: 11.6 years), after functional treatment (T1; mean: 12.8 years), and 2 years after orthodontic treatment (T2; mean: 18 years). Bilateral surface electromyographic activities of the anterior temporalis, posterior temporalis, masseter, and suprahyoid muscle areas were analyzed at rest and during clenching, swallowing, and mastication. Kinematic recordings of the mandibular maximum opening, lateral shift, right and left lateral excursions, and protrusion were evaluated. Results: Compared to T0, the left masseter activity during clenching was decreased at T1 but increased at T2, similar to the other evaluated muscles. The suprahyoid activity during swallowing was increased at T1 but decreased at T2. The masseter activity during mastication was increased at T1 and further increased at T2. The left and right lateral excursions and protrusion did not show significant changes throughout the experiment. Conclusions: Teuscher activator and subsequent fixed orthodontic treatment improved jaw muscle function; however, a long period was needed to attain complete neuromuscular adaptation.

** Key words:**Class II malocclusion, jaw muscles, mandibular kinematics, sEMG, Teuscher activator.

## Introduction

Angle Class II division 1 malocclusion, which has a high prevalence among different populations, is a very common problem encountered by orthodontists (1-4). Myofunctional alterations, such as reductions in the functional and postural activities of the jaw muscles, have been described in affected patients (5,6). Functional therapy with various orthodontic appliances is usually the first choice of treatment in cases of mandibular retrognatism. The main goal of functional appliance treatment of Class II malocclusion is to utilize the forces exerted by the masticatory muscles, tongue, cheeks, and lips to elicit neuromuscular changes, which can affect the activity of the masticatory muscles and the movement and position of the mandible (7,8).

Clinical studies have shown that activators, which have been widely used for many years, may influence the surface electromyo-graphic (sEMG) activity of the jaw muscles (9-13). In their examination of the sEMG activities of the masseter (MA) and anterior temporalis (AT) muscles, Uner et al. (10) observed increased activities of both muscles in the rest position and decreased activities in the maximum biting position at the beginning of treatment when an activator was used, whereas both muscle activities were decreased in the rest position at the end of treatment. No change was observed between the activities of MA and temporal muscles recorded at the beginning and at the end of this investigation without the activator. Ueda et al. (9) reported a considerable difference in the masticatory muscle activities induced by activator use during daytime and sleep. Ingervall et al. (12) Ingervall et al. (12) found that the sEMG activity of the posterior temporalis (PT) at the rest position increased after 6 months of activator treatment. However, the sEMG activity decreased after a period of observation, probably due to patient adaptation to the new mandible position.

Functional appliances cause neuromuscular alterations, and neuromuscular adaptation may not be complete within the 6 months of functional treatment. Thus, studies evaluating jaw muscle response after functional treatment should be performed over the long term, because a 6-month observation period may be insufficient to draw definitive conclusions (14). Moreover, the possibility of later adaptation effects during treatment with functional appliances is an important consideration.

Few studies have analyzed neuromuscular changes after treatment with the Teuscher activator, and no study has evaluated long-term changes with its use. We tested the hypothesis that functional treatment with the Teuscher-activator improves jaw muscle function. Therefore, the aim of this prospective, longitudinal, case-series study was to evaluate the sEMG activities of masticatory muscles and the kinematics of mandibular changes in children with Class II division 1 malocclusion treated by the Teuscher activator combined with high-pull headgear after functional treatment and at 2 years after orthodontic treatment completion.

## Material and Methods

-Subjects

Twenty-seven children diagnosed with angle Class II division 1 malocclusion and retruded mandible were consecutively recruited from the Faculty of Odontology at the graduate orthodontic clinic of the University. Children were selected for the study according to the following inclusion criteria: skeletal and dental Class II division 1 malocclusion, dolichofacial pattern, retrognathic mandible, bilateral Class II molars of at least ½ cusp, no unilateral posterior cross-bite, hand-wrist radiographic stage before the peak of the pubertal growth spurt, and Caucasian origin. Subjects were excluded if they had temporomandibular joint noises at clinical examination (open-close), capsular or muscle pain on palpation, a history of neuromuscular disease or disease affecting neuromuscular performance, trauma in the dentofacial region, systemic joint disease, previous orthodontic treatment, or were taking systemic medication, such as steroids.

Patients were evaluated before orthodontic treatment (T0, mean age: 11.6 years), after functional treatment with the Teuscher activator (T1, mean age: 12.8 years), and at 2 years after completion of orthodontic treatment with fixed appliances (T2, mean age: 18 years). All patients and their parents agreed to participate by signing an informed consent form that had been approved by the Ethical Committee of our Institution.

-Orthodontic treatment

Patients received functional treatment with the Teuscher activator with extraoral high-pull headgear. For bite registration, each patient was asked to protrude his or her mandible as far as possible. Four millimeters were subtracted from this position, and the construction bite was registered. The construction bite was standardized for all patients with the George gauge (Great Lakes Orthodontics, Tonawanda, NY), which automates bite registration for functional orthodontic appliances simply and accurately. The construction bite was not taken in an edge-to-edge position, which supposes a different advancement of the mandible for each patient, depending on the overjet. At the start of the treatment, each subject was instructed to wear the activator for at least 14 hours daily. To assess the degree of compliance, patients were asked to register their daily wearing times on a special form. The mean functional treatment time was 1.1 years. After treatment with the activator had finished, orthodontic treatment was continued with fixed appliances (0.018-inch-slot conventionally ligated Hilgers’ edgewise brackets system; Ormco, Glendora, CA). The mean orthodontic treatment time with fixed appliances was 2.6 years, followed by retention for 6 months with a maxillary removable circumferential Hawley-type retainer and a mandibular flexible spiral wire canine-to-canine lingual retainer bonded to all 6 anterior teeth. After the patients had completed their orthodontic treatment protocols, they were followed for 2 years out of retention.

-Electromyography study

The electromyography study was performed with an EM2 electromyograph (K6-I Diagnostic System, Myotronics-Noromed, Kent, WA), which has 8 channels, a frequency bandwidth response of 45-430 Hz per channel, and allows 4 pairs of muscles to be tested simultaneously. The EM2 was interfaced with a computer. Disposable 10-mm-diameter Ag/AgCl bipolar surface electrodes (Duo-Trode Myotronics-Noromed) were positioned, with an interelectrode distance of 21 ± 1 mm, on the muscle bellies parallel to muscle fibers, according to a previous protocol (15). Simultaneous bilateral (right and left) sEMG activities from the AT, PT, MA, and suprahyoid (SH) muscle areas were recorded at the mandibular rest position, during swallowing, and during mastication. The sEMG activities from the bilateral AT and MA muscles were obtained during maximal voluntary clenching (MVC) in maximal intercuspation. Asymmetry, activity, and torque indices(16,17) were calculated for each muscle at rest and during MVC. The MA/TA ratio during MVC was also recorded. To assess the reproducibility of sEMG data, 5 subjects underwent 4 trials over 4 days according to a tested experimental protocol (15).

-Kinesiography study

Mandibular movements were recorded with a kinesiographic computer system (K6, Myo-tronics, Seattle, WA). Sensor placement, instrument alignment and calibration, and recording were performed according to previously described protocols (18-20). Mandibular movements were recorded during maximum excursions (opening-closing, protrusion, and right and left lateral). As described in a previous study (19), the reproducibility of the kinesiographic recordings was tested by comparing the results of 2 consecutive measurements of 10 randomly selected subjects.

-Statistical analysis

The SPSS 11.0 software package (SPSS Inc, Chicago, IL) was used for statistical analysis. After establishing normality by the Shapiro-Wilks test, data were compared at T0 vs. T1 vs. T2 with 1-way repeated measures ANOVA followed by the Tuk-ey-Kramer multiple-comparison test. The ANOVA for repeated measures test was used to test the reproducibility of the EMG measurements. The reproducibility of the kinesiographic data was tested by the paired t-test. Differences with P ≤ 0.05 were considered statistically significant.

## Results

The reproducibility of sEMG recordings was assessed from repeated measurements (4 trials) from different subjects performed over different days. ANOVA revealed no systematic differences. A paired Student’s t-test revealed no systematic differences between the first and second data collection sessions for the kinesiographic recordings.

-Muscle activity at rest position

( Table 1) shows the mean values and standard deviations of the electric potentials recorded in the 8 examined muscle areas at the 3 time points. Significant changes in the sEMG activities were found for all muscle areas. Posthoc comparisons ( Table 2) showed significantly higher sEMG values of the left AT and right SH muscle areas after treatment (T0-T1), which reached values similar to the corresponding contralateral muscles. We also observed significant increases in the resting sEMG activities of the AT, PT, and MA areas and a significant decrease in the activity of the SH area from the end of treatment completion to the end of the observation period (T1-T2). No differences were found for any of sEMG indices throughout the study ( Table 3). The activity index became more negative from T1 to T2, reaching a final value of -20.07 ± 25.07. reflecting that jaw position is maintained by using more AT than MM muscles.

**Table 1:**
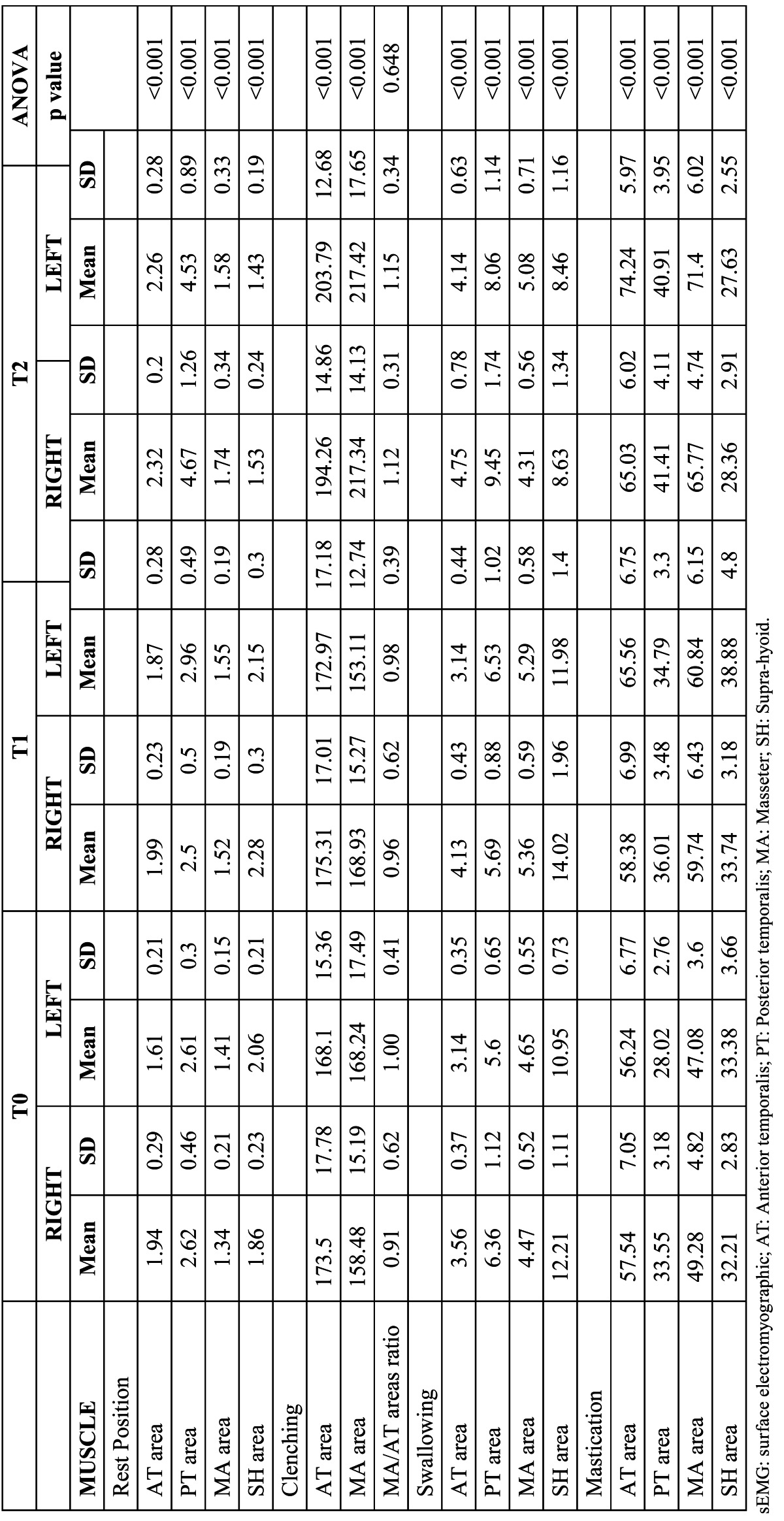
Comparisons of mean values of sEMG activity (µV) at rest position, during clenching, swallowing and mastication at T0, T1 and T2 (One-way ANOVA for repeated measurements).

**Table 2:**
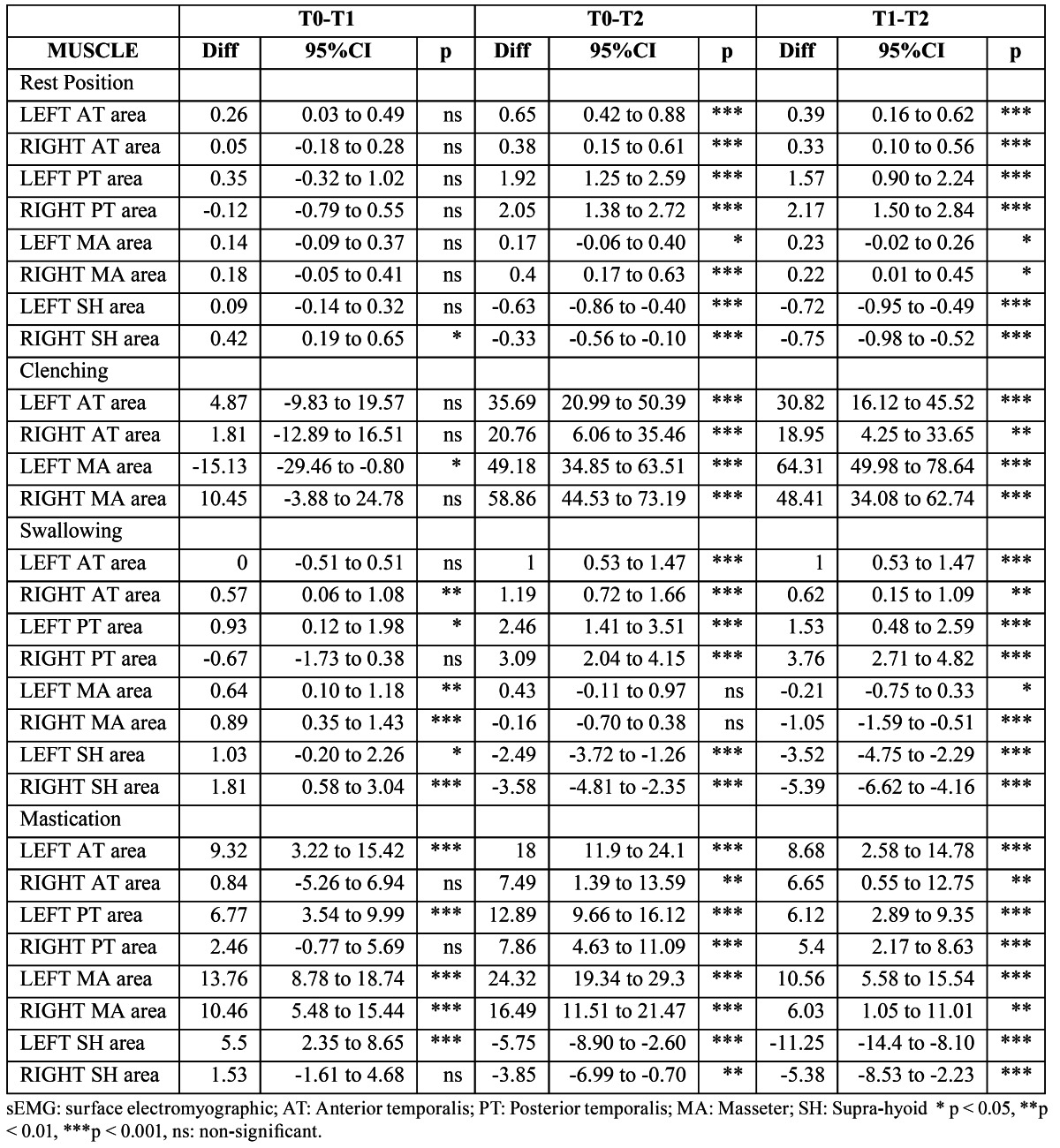
Mean differences (T0 vs. T1, T0 vs. T2, T1 vs. T2) of sEMG activity (µV) at rest position and during clenching, swallowing and mastication (Tukey post-tests performed after one-way ANOVA shown in Table 1).

**Table 3:**
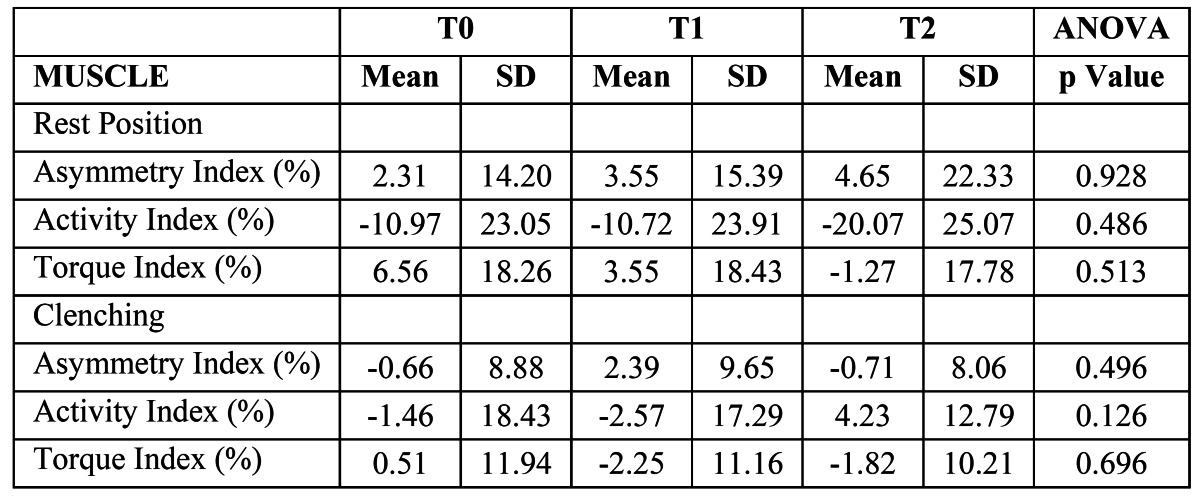
Comparisons of mean values of Asymmetry, Activity and Torque Indices (%) at rest position and during clenching at T0, T1 and T2 (One-way ANOVA for repeated measurements).

-Muscle activity during MVC

( Table 1) lists the sEMG values of the AT and MA muscle areas during MVC. The activity of the left MA area decreased significantly after treatment, but this reduction was not stable. The activity of the left MA area increased significantly, similar to the other evaluated muscle areas, from T1 to T2 ( Table 2). The ratio of the MA/TA areas and the EMG indices ( Table 3) remained unchanged over time.

-Muscle activity during swallowing 

Right AT and left PT activities increased significantly after treatment and continued to increase through the end of the study. During the observation period after functional treatment (T1–T2), the left and right AT and PT muscle areas showed increased activities. In contrast, the left and right MA and SH muscles showed increased activities after treatment, but this increment was unstable and decreased during the observation period ( Table 1, Table 2).

-Muscle activity during mastication

A significant increment in the left AT and PT and both MA muscle area activities was found after treatment, which continued to increase after the observation period. At the end of the study, bilateral AT, PT, and MA muscle areas showed highly significant sEMG values. The left SH activity increased after treatment but decreased during the observation period, and both SH areas showed lower sEMG values at the end of the study ( Table 1, Table 2).

-Kinesiographic results

( Table 4) presents the kinesiographic data of the mandibular maximum opening, lateral shift, right and left lateral excursions, and protrusion for the 3 study periods. A temporary non-significant increase of maximum opening was observed after treatment with the Teuscher activator (from 36.59 mm to 41.46 mm). Tukey post-tests performed after one-way ANOVA for repeated measurements shown in ( Table 4) showed significant differences only for the mandibular lateral shift: the initial lateral shift (0.45 ± 1.16 mm) increased after functional treatment (1.33 ± 0.81 mm) (p<0.05), but decreased during the observation period (to 0.56 ± 0.45 mm) (p<0.05).

**Table 4:**
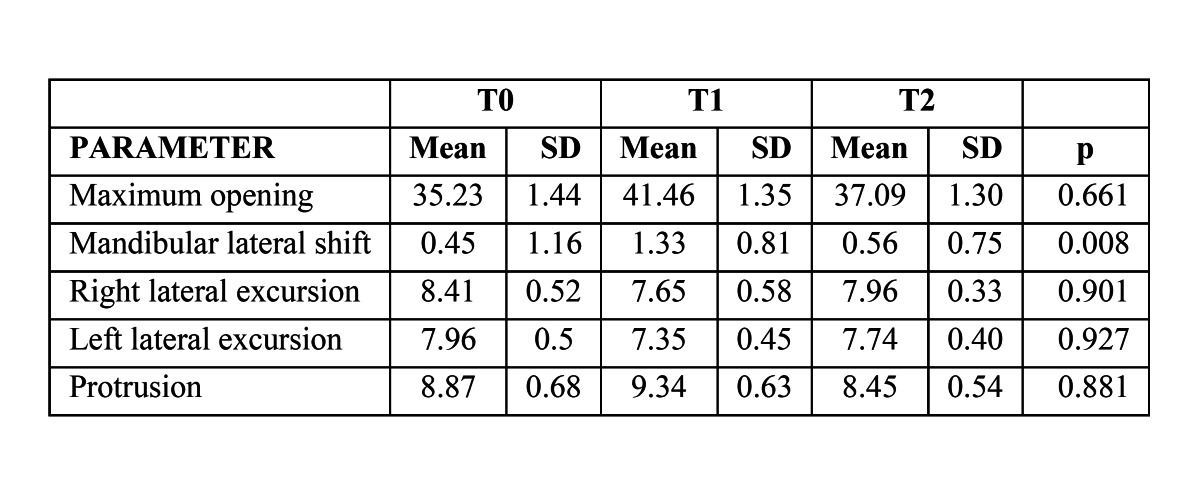
Comparisons of kinematic measurements (mm) of maximum opening, mandibular shift, right and left lateral excursions and protrusion at T0, T1 and T2 (One-way ANOVA for repeated measurements).

## Discussion

We analyzed the sEMG activity of the jaw muscles and used kinesiography to assess longitudinal changes in the mandibular rest position and during clenching, swallowing, mastication, maximum opening, lateral shift, lateral excursion, and protrusion in children with Class II division 1 malocclusion after functional treatment with the Teuscher-activator and at 2 years after retention. Our working hypothesis was accepted since activator use improved jaw muscle function.

One limitation of this study was the lack of a longitudinal control group with normal occlusion throughout all phases of the study. However, there are few, if any, published studies including normative sEMG values of the jaw muscles and kinematics of the mandible in growing children.

-Muscle activity at rest position

A more symmetrical muscular condition was achieved after functional treatment. Similar to Ingervall et al. (13), Ingervall et al. (13), we did not find a decrease in the activity of the PT muscles after functional treatment, although such a decrease has been described as a sign of forward displacement of the mandible during treatment with a functional appliance (21).

The PT muscle showed the highest sEMG values in the rest position throughout the study, reflecting the role of the PT muscles in the stabilization and positioning of the mandible at rest in patients with Class II division 1 malocclusion, as in other studied samples (22). In contrast, the MA muscle presented the lowest activity. The activity index showed a predominance of AT over MA in the rest position before treatment, supporting the important role that AT muscles play in positioning the mandible in Class II malocclusion in normal young people (16) and young adults (23). Similarly, the activity index was more negative at the end of the observation period, reflecting that the jaw position at this time was maintained more by the AT than by the MA muscles. During the 2-year observation period after orthodontic treatment was completed (T1–T2), the AT, PT, and MA muscles all showed increased activities, whereas the SH showed decreased activity. This finding reflects good neuromuscular adaptation of the mandible to the rest position, new skeletal and occlusal conditions, and general growth changes.

-Muscle activity during MVC

We observed a decrease in the sEMG activity of the left MA during MVC after functional treatment. This finding was probably due to occlusal instability or a lack of occlusal contacts of teeth in the posterior region, caused by the changed tooth positions and the intermaxillary relationship after functional treatment. Ingervall et al. (12) Ingervall et al. (12) and Ingervall et al. (13) Ingerval et al. (13) found a decrease in the maximal activity of the MA muscles after functional treatment with an activator, and Aggarwal et al. (14) made the same observation after treatment with the Twin-block appliance. During the observation period, we found that the MA activity increased significantly, as did the activities of other evaluated muscle areas (right MA and both AT muscles). This finding reflects improvement of the occlusal conditions (i.e., contact quality and stability).

Before treatment with the functional appliance, the activity of AT was higher than (right AT) or similar to (left AT) the activity of the homolateral MA muscles. Similar findings have been found previously in Class II division I malocclusion samples (6,24). After functional treatment, the AT muscles showed higher sEMG values than the MA muscles. Although no significant difference was found in the activity indices or in the MA/AT area ratios throughout the study, there was a slight tendency toward predominance of MA over AT as the activity index moved from negative (AT predominance) at T0 and T1 to positive (MA predominance) at T2, approaching the situation in normo-occlusive young people (16,25). This improvement in the MA sEMG values could be explained by improvements in the occlusal conditions after completion of the treatment and observation period. The long-term changes might also be attributable to the growth and development of the children, particularly in more advantageous conditions.

-Muscle activity during swallowing 

Interestingly, children with class II division 1 malocclusion showed high sEMG values in the SH muscles during swallowing. This finding could be attributed to the increased overjet that forced the tongue to move forward during swallowing, such as occurs in immature swallowing (26). However, after functional treatment, the SH activities were increased, even though the overjet was reduced. At 2 years after orthodontic treatment completion, the activities in the SH muscle were significantly decreased. This finding reflects normalization in the SH function, probably related to a more mature swallowing. Overall, these results might indicate that neuromuscular changes related to the normalization require a long period after functional treatment in which to occur. As a reflection of this functional improvement after a long time period, the AT and PT muscles also showed increased activities, whereas the MA activities decreased during the T1–T2 time interval.

-Muscle activity during mastication

From a functional perspective, the most important finding was that the MA sEMG activities during mastication increased significantly after treatment with the Teuscher activator (13.76 µV mean left MA, 10.46 µV mean right MA) and increased further at 2 years after orthodontic treatment completion. At the end of the study, the AT and PT muscles showed significantly higher activity values. In contrast, the activities of the SH muscle areas were decreased after the observation period, similar to their values in the rest position and during swallowing. Therefore, the functional capacity of the jaw muscles during mastication improved after functional treatment and continued to improve during the years after orthodontic treatment was completed.

-Kinesiography

Before treatment, patients with Class II division 1 malocclusion showed a maximum vertical opening of 35.23 ± 1.44 mm, similar to the value reported by Ferrario et al. (27) in normal young people (36.59 mm). The observed temporary increase seen after treatment with the Teuscher activator (41.46 ± 1.35 mm) could be explained by the increased laxity of the TMJ ligaments produced by the functional appliances. On the other hand, Thüer et al. (8) did not find changes in the maximum vertical opening during activator treatment. The mean mandibular lateral shift experienced a transient increment after treatment (from 0.45 to 1.33 mm) but decreased to near its original value during the observation period (0.56 mm). These results can be interpreted as a consequence of the remodeling processes that occur at the TMJ. Some studies (28,29) have reported a slight lateral displacement of the mandible during opening and closing movements in normo-occlusive young patients, which is considered physiological.

The left and right lateral excursions and protrusion did not show significant changes after functional treatment or after the observation period. Petit et al. (30) Petit et al. (30) described a reduction in the magnitude of the maximal protrusion as a sign of forward mandibular positioning during activator treatment. From this perspective, our patients did not experience a forward mandibular position after Teuscher activator treatment.

## Conclusions

Teuscher activator with extraoral high-pull headgear and subsequent fixed orthodontic treatment improved jaw muscle function. Our findings suggest a good adaptability of the jaw muscles to the newly achieved skeletal and occlusal conditions after Teuscher activator treatment, although a long period was needed to reach complete neuromuscular adaptation to these changes. Longitudinal studies including a normo-oclusive–matched control group are needed to clarify which changes may be specifically attributed to the functional treatment.
